# Supplementary material for: MetaRibo-Seq measures translation in microbiomes
Source: Nat Commun. 2020 Jun 29;11:3268. doi: 10.1038/s41467-020-17081-z (PMC7324362; doi:10.1038/s41467-020-17081-z)
Supplement: Supplementary file 10 — Supplementary Data 7 [file 41467_2020_17081_MOESM10_ESM.zip › File2/Confidence_High_Taxonomy/90869_out.krona.html]

Javascript must be enabled to view this page.

members
magnitude
magnitudeUnassigned
count
unassigned
taxon
rank

90869\_out

4

superkingdom
2
4

4
1239
phylum

186801
4
class

order
4
186802

family
1
31979

genus
1
1485


SRS019445\_contig\_number\_contig-100\_32463.79628
species
1
59620

family
1
186806

1
1730
genus

2292352
1

SRS048870\_contig\_number\_15447
species

186803
2
family

1
1164882
genus

617123
1

SRS056896\_contig\_number\_15924
species

genus
33042
1

1
410072

SRS148424\_contig\_number\_contig-100\_10028.109859
species
